# Selective Interaction of Syntaxin 1A with KCNQ2: Possible Implications for Specific Modulation of Presynaptic Activity

**DOI:** 10.1371/journal.pone.0006586

**Published:** 2009-08-13

**Authors:** Noa Regev, Nurit Degani-Katzav, Alon Korngreen, Adi Etzioni, Sivan Siloni, Alessandro Alaimo, Dodo Chikvashvili, Alvaro Villarroel, Bernard Attali, Ilana Lotan

**Affiliations:** 1 Department of Physiology and Pharmacology, Sackler Faculty of Medicine, Tel-Aviv University, Ramat-Aviv, Israel; 2 Mina & Everard Goodman Faculty of life Sciences, Bar-Ilan University, Ramat-Gan, Israel; 3 Unidad de Biofísica, CSIC-Universidad del País Vasco (UPV)/Euskal Herriko Unibersitatea, UPV, Leioa, Spain; UMR CNRS 5226 - Université Bordeaux 2, France

## Abstract

KCNQ2/KCNQ3 channels are the molecular correlates of the neuronal M-channels, which play a major role in the control of neuronal excitability. Notably, they differ from homomeric KCNQ2 channels in their distribution pattern within neurons, with unique expression of KCNQ2 in axons and nerve terminals. Here, combined reciprocal coimmunoprecipitation and two-electrode voltage clamp analyses in *Xenopus* oocytes revealed a strong association of syntaxin 1A, a major component of the exocytotic SNARE complex, with KCNQ2 homomeric channels resulting in a ∼2-fold reduction in macroscopic conductance and ∼2-fold slower activation kinetics. Remarkably, the interaction of KCNQ2/Q3 heteromeric channels with syntaxin 1A was significantly weaker and KCNQ3 homomeric channels were practically resistant to syntaxin 1A. Analysis of different KCNQ2 and KCNQ3 chimeras and deletion mutants combined with *in-vitro* binding analysis pinpointed a crucial C-terminal syntaxin 1A-association domain in KCNQ2. Pull-down and coimmunoprecipitation analyses in hippocampal and cortical synaptosomes demonstrated a physical interaction of brain KCNQ2 with syntaxin 1A, and confocal immunofluorescence microscopy showed high colocalization of KCNQ2 and syntaxin 1A at presynaptic varicosities. The selective interaction of syntaxin 1A with KCNQ2, combined with a numerical simulation of syntaxin 1A's impact in a firing-neuron model, suggest that syntaxin 1A's interaction is targeted at regulating KCNQ2 channels to fine-tune presynaptic transmitter release, without interfering with the function of KCNQ2/3 channels in neuronal firing frequency adaptation.

## Introduction

The voltage-dependent M-type potassium current (M-current) is a subthreshold, slowly activating and noninactivating voltage-gated potassium current that is thought to stabilize membrane potential and control neuronal excitability by limiting repetitive firing [1]–[4].

The heterotetrameric KCNQ2/KCNQ3 channel complex, which belongs to the KCNQ family of voltage-dependent K^+^ channels, has been identified as the main molecular correlate of the M-channel [5]–[7]. KCNQ2 and KCNQ3 are coexpressed on the cell bodies and dendrites of many hippocampal and cortical neurons [2], [7]. Also, the initial segment of several neurons in the hippocampus, neocortex, brainstem and striatum show colocalization of KCNQ2 and KCNQ3 expression [8], [9]. Notably, KCNQ2, but not KNCQ3, subunits are expressed presynaptically on axons and nerve terminals, where they might regulate action potential propagation or neurotransmitter release [8], [10].

Modulation of the M-current has profound effects on brain excitability. Inhibition of M-channels by muscarinic agonist and other neurotransmitters enhances action-potential firing in central and autonomic neurons [2], [11], [12]. In addition, a number of neuropeptides [1], [2], [13], [14], and many types of second messengers [3], [12], [15]–[19], have been implicated in M-current modulation. Recent studies have shown that calmodulin (CaM) binds to the KCNQ2 and KCNQ3 C termini and may function as an auxiliary channel subunit [17], [20], [21].

A new class of proteins capable of interacting with Kv channels is made up of the SNARE (soluble *N*-ethylmaleimide-sensitive fusion protein attachment protein receptor) proteins: the plasma membrane (PM) syntaxin 1A, SNAP-25 and the vesicle-associated membrane protein 2 (VAMP2). These proteins form the minimal molecular complex, common to all secretory processes, controlling the docking of synaptic vesicles and their fusion with the presynaptic membrane [22]–[26]. In particular, syntaxin 1A has been well-established by our and other laboratories to directly bind to and modulate three Kv channels: Kv1.1 [27], Kv2.1 [28]–[30] and Kv2.2 [31]. These interactions were shown to be mediated by the cytoplasmic termini of the channels.

In the present study, we demonstrate that syntaxin 1A also associates with KCNQ2 subunits in the brain, leading to modulation of KCNQ2 homomeric channel gating in oocytes. Interestingly, these interactions are mediated via the C-terminal helix A of the channel, which constitutes part of the CaM-binding site [17], [20], [21]. Colocalization of KCNQ2 and syntaxin 1A at synaptic terminals suggests a role for their interaction in vesicle release, similar to the recently identified novel role for the syntaxin 1A-Kv2.1 interaction in the enhancement of dense-core vesicle release [32], [33].

## Materials and Methods

### Constructs and antibodies

The primary antibodies used were KCNQ2-C terminus, KCNQ3-C terminus (Alomone Labs, Jerusalem, Israel) and monoclonal anti-HPC-1 (Sigma Israel, Rehovot, Israel). cDNAs and mRNAs of the chimeric channels with different transmembrane segments were described [34], [35]. KCNQ2-HA (tagged with Ha epitope in the extracellular loop that contacts transmembrane domains S1 and S2) was kindly provided by Thomas Jentsch (Zentrum für Molekulare Neuropathobiologie, Hamburg, Germany). KCNQ2-YFP was constructed by subcloning of native KCNQ2 into PGEMHJ vector containing eYFP at CT position, between two *XbaI* restriction sites. DNAs of KCNQ2 and KCNQ3 fragments to create GST fusion proteins were described [35]. Enzymes were purchased from Promega (Madison, WI) or MBI Fermentas (Vilnius, Lithuania).

### Oocyte culture

Oocytes of *Xenopus laevis* were prepared as described [36]. Oocytes were injected (50 nl per oocyte) with 5 ng/oocyte KCNQ2/1 ng/oocyte KCNQ2+KCNQ3 (1∶1 ratio)/1 ng/oocyte KCNQ3*+KCNQ3/2.5 ng/oocyte KCNQ2-HA/17.3 ng/oocyte KCNQ2-YFP, with or without 0.75 ng/oocyte syntaxin 1A mRNAs for electrophysiological and imaging experiments.

### Electrophysiological recordings in oocytes

Two-electrode voltage-clamp recordings were performed as described [37]. Current-voltage relationships were obtained by depolarizing steps from −85 mV to +20 mV by increments of 15 mV. Net current was obtained by subtracting the scaled leak current elicited by a voltage step from −100 to −110 mV. Oocytes with a leak current of >3 nA/1 mV were discarded.

### Immunocytochemistry and imaging in oocytes

To visualize the HA tag, whole oocytes expressing KCNQ2-HA were fixated in 4% formaldehyde (37%) in Ca-free ND96 solution for 15 min, 3 days after the injection of mRNA. . Blocking of nonspecific binding sites was done by 5% skim milk for 1 h. Then the oocytes were incubated for 1 h with the mouse monoclonal IgG2a antibody against HA (Santa Cruz Biotechnology), diluted 1∶400 in 2.5% skim milk. Residual antibody was washed out with 2.5% skim milk three times, 5 min each. This was followed by 1 h incubation with the secondary antibody (Alexa-conjugated anti–mouse IgG, 1∶400; Jackson ImmunoResearch Laboratories) in dark. Free secondary antibody was then washed out with Ca-free ND96. Oocytes were placed in a chamber with a transparent bottom, and fluorescence imaging was performed with LSM 510 (×20 objective, zoom = 2, pinhole 3 Airy units). Alexa was excited at 594 nm and the emitted light was collected using long-pass (LP) 615-nm filter. Imaging of KCNQ2-YFP channels was performed with LSM 510 (×20 objective, zoom = 2, pinhole 3 Airy units). eYFP was excited at 514 nm and the emitted light was collected using long-pass (LP) 615-nm filter. Because of the strong auto fluorescence of the vegetal pole and/or from the intracellular milieu, fluorescence signal was collected from dark animal pole [38]. All images were obtained from optical slices from the animal hemisphere close to oocyte's equator. Quantification of all the images was done using Zeiss LSM software. The fluorescent signals were analyzed by averaging the signal obtained from four standard circular regions of interest as well as four background regions. Net fluorescence intensity per unit area was obtained by subtracting the background signal measured in native oocytes [39]. In all confocal imaging procedures, care was taken to completely avoid saturation of the signal. In each experiment, all oocytes from the different groups were studied using constant LSM settings.

### Immunoprecipitation (IP) and immunoblotting (IB) in synaptosomes

We used fresh synaptosomes that had been stored in aliquots at −80°C and thawed once. IP was performed as described [22]. Briefly, antibodies were prebound to protein A-Sepharose beads (Zymed, South San Francisco, CA) in HKA buffer (50 mM HEPES-KOH, pH 7.4, 140 mM K-acetate, 1 mM MgCl_2_, and 0.1 mM EGTA) supplemented with 0.1% gelatin and 0.1% bovine serum albumin (BSA). Synaptosomes were washed gently twice and solubilized for 1 hr at 4°C in IP buffer containing HKA buffer with the addition of 2% freshly prepared y3-[(3-cholamidopropyl)dimethylammonio]-1-propanesulfonic acid (CHAPS). Protease inhibitors (10 µg/ml aprotonin, leupeptin, and pepstatin; Boehringer Mannheim) were added to the IP buffer. After overnight incubation of the prebound beads (4°C) with solubilized synaptosomes, the bound proteins were thoroughly washed (in IP buffer with only 0.2% CHAPS), separated by SDS-PAGE, and subjected to Western blot analysis using the ECL detection system (Amersham, Buckinghamshire, UK). Special precautions were taken to avoid nonspecific interactions with syntaxin 1A adhering to protein A-Sepharose beads. Such adhesion was minimized by including gelatin in the experiment and 5% glycerol in the final washing step.

### “Pull-down” of synaptosomal proteins

GST fusion proteins immobilized on glutathione–Sepharose beads were incubated with rat brain synaptosomes (P2 fraction) in HKA buffer with 2% CHAPS and a mixture of protease inhibitors (Boehringer Mannheim) at 4°C for 12 hr. Samples were washed four times with HKA containing 0.1% Triton X-100, then boiled for 10 min in SDS sample buffer, electrophoresed (12% polyacrylamide gel), immunoblotted and processed as described above. ECL signals were quantified with TINA software (Budapest, Hungary).

### Immunoprecipitation in oocytes

Oocytes were subjected to immunoprecipitation as described [37]. Briefly, immunoprecipitates from 1% Triton X-100 homogenates of either plasma membranes (PMs) or internal fractions (IFs) [separated mechanically, as described [40]] or whole oocytes were analyzed by SDS–PAGE (usually on gradients of 8% to separate KCNQ3 from the lower band of KCNQ2). Digitized scans were derived by PhosphorImager (Molecular Dynamics, Eugene, OR) and relative intensities were quantitated by ImageQuant.

### In vitro binding of GST fusion proteins with Syntaxin 1A

The fusion proteins were reacted with syntaxin 1A as described [35]. Briefly, purified GST fusion proteins immobilized on glutathione–Sepharose beads were incubated with the lysate containing ^35^S-labeled syntaxin 1A [syntaxin 1A translated on the template of *in vitro* synthesized RNAs using a translation rabbit reticulocyte lysate kit (Promega) according to the manufacturer's instructions] with gentle rocking. After washing, the GST fusion proteins were eluted with 20 mM reduced glutathione in 30 µl elution buffer (120 mM NaCl, 100 mM Tris–HCl, pH 8) and then subjected to SDS–PAGE (12% polyacrylamide).

### Immunocytochemistry in hippocampal neurons

Experiments were performed on dissociated cultures from hippocampus of E18 rat embryos. The hippocampal neurons were grown in culture for 10 to 14 days on 13-mm-diameter coated glass coverslips in 24-well plates. Cells were carefully rinsed for 10 min in phosphate-buffered saline (PBS), and the neurons were subsequently fixed for 20 min in 4% paraformaldehyde in PBS. After extensive washes in PBS, the cells were blocked and permeabilized by incubation with 10% horse serum (HS) in PBS containing 0.04% Triton X-100. Cells were then washed for 10 min in PBS containing 3% HS. Neurons were incubated at 4°C overnight with two or more of the following primary antibodies diluted in PBS containing 3% HS: a goat polyclonal antibody to KCNQ2 (N19, 1∶150; Santa Cruz Biotechnology Inc., Santa Cruz, CA), a mouse monoclonal anti-syntaxin 1A (1∶2500; Sigma-Aldrich, St. Louis, MO) and a rabbit polyclonal anti-VAMP-2 (Synaptobrevin 2; 1∶800, Alomone Laboratories, Jerusalem, Israel). After a wash in PBS, cells were incubated for an hour at room temperature with secondary antibodies: cy2-conjugated anti-mouse IgG (1∶150; Jackson ImmunoResearch Laboratories Inc., West Grove, PA), rhodamine red X-conjugated anti-goat IgG (1∶200; Jackson ImmunoResearch Laboratories Inc.) and/or Alexa fluor 633 ant-rabbit (1∶2000; Invitrogen, Carlsbad, CA). Neurons were viewed and digital images taken using a Zeiss LSM 410 confocal microscope. Colocalization of markers was analyzed with Image-Pro Plus 4.5 software MediaCybernetics, Inc., Silver Spring, MD, USA) using Pearson's correlation. All data were expressed as mean±SEM.

### Computational Simulation

All simulations used the NEURON (versions 5.9 and 6.0) simulation environment [41], with an integration time step of 25 µs. . We used the default NEURON implementation of the classical Hodgkin-Huxley model of the giant squid axon [42]. This model contained two conductances, voltage-gated sodium and voltage-gated potassium conductances. This classical model generated train of evenly spaced action potentials when depolarizing current was injected via a simulated electrode. On top of this simple model we inserted a kinetic model of KCNQ2 or KCNQ2/3 that was based on data reported in this work. Ion channel models of the KCNQ conductances were implemented using the NMODL extension of the NEURON simulation language [43]. In order to simulate an after depolarizing potential (ADP) we inserted a t-type Ca^2+^ conductance model in addition to the other conductances [44], [45].

The kinetics of the KCNQ2 and KCNQ2/3 conductances were extracted from the recordings presented in this manuscript using double exponential curve fitting to the current traces recorded in the voltage-clamp mode. The curve fitting revealed that the slow time constant and fast time constant of activation contributed equally to the current of both KCNQ2 and KCNQ2/3. Thus, the model of the two currents used the same voltage dependence of activation and two time constants assuming a single activation gate according to the Hodgkin-Huxley formalism [42]. The equations describing the model are similar for all conductances modeled in this work:
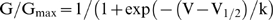


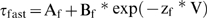


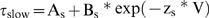



Where G/G_max_ is the conductance normalized to its maximal value, V is membrane potential; V_1/2_ is the voltage at which the conductance is half-maximal and k is the slope factor. The kinetic parameters for all the conductances were summarized in Table 1.

**Table 1:** Values of kinetic parameters used in simulating the physiological effect of the conductances investigated in this paper.

	V_1/2_ (mV)	k (mV)	A_f_ (ms)	B_f_ (ms)	z_f_ (1/mV)	A_s_ (ms)	B_s_ (ms)	z_s_ (1/mV)
**KCNQ2**	−50	5.8	68	18	0.069	290	85	0.071
**KCNQ2+syx**	−50	5.8	100	76	0.040	580	83	0.075
**KCNQ2/Q3**	−50	5.8	143	168	0.061	636	277	0.067

The values were extracted by exponential curve fitting of the voltage dependence displayed by the activation time constant extracted from voltage-clamp recordings. The parameters relate to equations 1–3 in the text.

*Statistical analysis*. Data are presented as means±SEM. Student's *t* test was used to calculate the statistical significance of differences between two populations. Graphical presentation, fitting and statistical analysis were performed using SigmaPlot with integrated statistical module of SigmaStat (Systat Software, Inc., San Jose, CA, USA).

## Results

### Syntaxin 1A strongly associates with KCNQ2 subunits but not with other KCNQ family members in *Xenopus* oocytes

**Figure 1:**
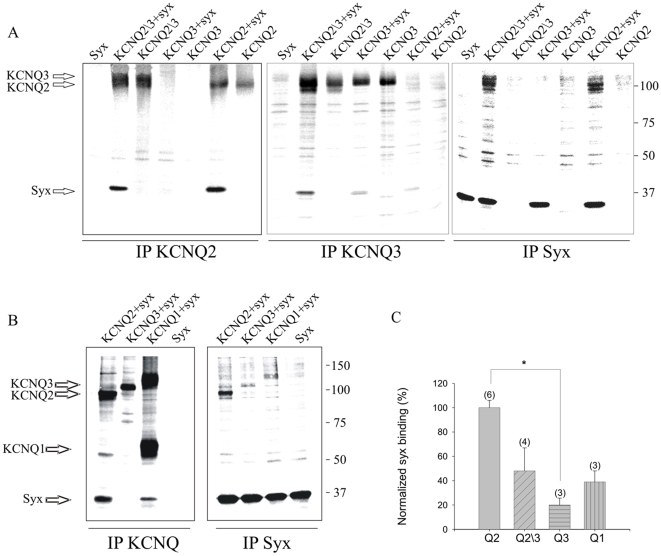
Syntaxin 1A interacts physically with KCNQ2, KCNQ2\3 and KCNQ1, but hardly interacts with KCNQ3 in oocytes. A, Digitized Phosphorimager scan of SDS-PAGE analysis of [^35^S] Met/Cys-labeled channels (KCNQ2; KCNQ2/3 and KCNQ3) and syntaxin 1A (*syx*) proteins coprecipitated by the corresponding antibodies from 1% Triton X-100 homogenates of whole oocytes, that were injected with the channels mRNA alone or with syntaxin 1A mRNA alone or coinjected with the channels and syntaxin 1A mRNAs (as indicated above the lanes). The protein samples were analyzed on an 8% gel. Arrows indicate the relevant proteins. B, KCNQ3 and KCNQ1 channels do not interact with syntaxin 1A as strongly as KCNQ2 in oocytes. *Left panel*: The channels and syntaxin proteins coprecipitated by the corresponding antibodies (as indicated below the lanes). *Right panel*: Reciprocal coimmunoprecipitation in oocytes from the same experiment, carried out using a monoclonal syntaxin 1A antibody (IP syx). C, Interaction of KCNQ2, KCNQ2\3, KCNQ3 and KCNQ1 with syntaxin 1A. Bars depict ratios (quantified by ImageQuant) of syntaxin to the channels, precipitated by the corresponding channel antibodies. Numbers in parentheses refer to number of oocyte batches. *p<0.05.

Following our previous studies, in which we characterized syntaxin 1A's interactions with Kv channels [29]–[31], [46], [47], we studied the interaction of syntaxin 1A with KCNQ2, KCNQ2/3 and KCNQ3 channels in the heterologous expression system of *Xenopus* oocytes, where biochemical and electrophysiological analyses can be performed simultaneously. First, we carried out a comparative examination of the physical interactions of syntaxin 1A with the channels by performing reciprocal coimmunoprecipitation analysis, using antibodies against KCNQ2, KCNQ3 and syntaxin 1A, in oocytes from a single batch coexpressing the subunits with syntaxin 1A. Syntaxin 1A associated with KCNQ2, to a lesser extent with KCNQ2/3 and only weakly with KCNQ3 (Fig. 1A). Further analysis of the specificity of the syntaxin 1A interaction with KCNQ family members showed that the interaction with KCNQ1 is weaker than that with KCNQ2 (Fig. 1B). Quantification over several similar experiments of the intensity ratios of coprecipitated syntaxin 1A to the different channel subunits, coexpressed in the same cells, showed that the amount of syntaxin 1A associated with KCNQ2 was ∼two-, five- and threefold larger than that with KCNQ2/3, KCNQ3 and KCNQ1, respectively (Fig. 1C).

#### Syntaxin 1A modulates primarily the KCNQ2 currents

**Figure 2:**
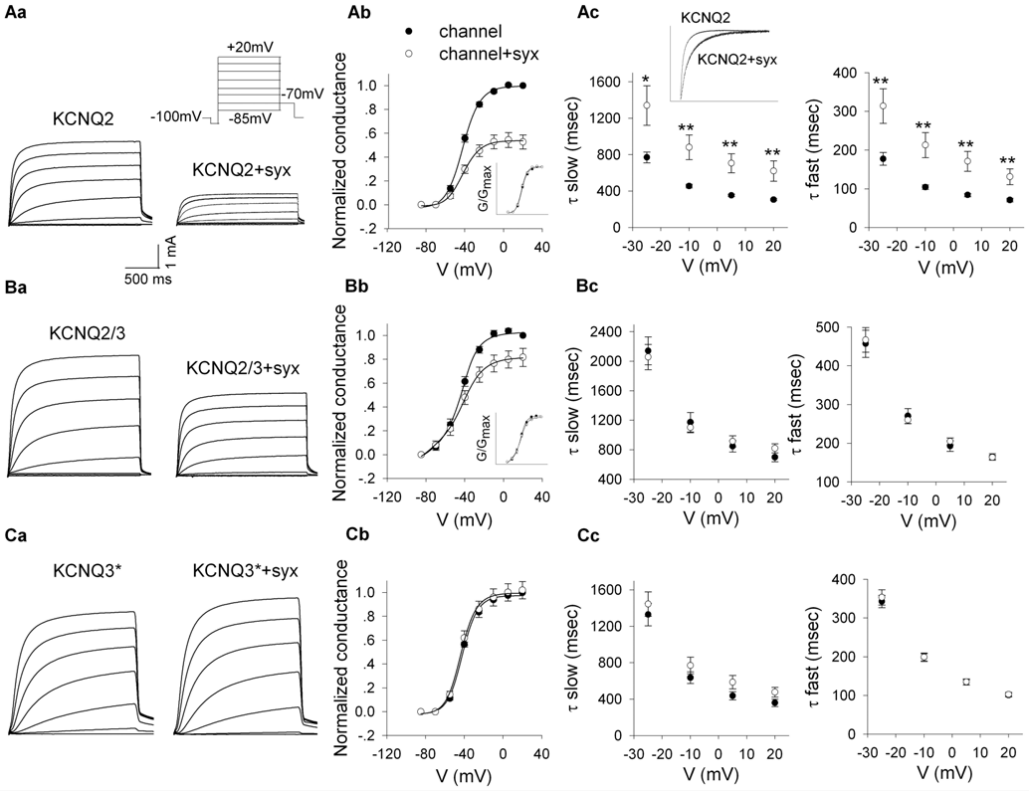
Syntaxin 1A modulates primarily the KCNQ2 currents. A, Syntaxin 1A (syx) reduces current amplitudes of KCNQ2 (Aa) and KCNQ2\3 (Ba) channels, expressed in Xenopus oocytes, but not those of KCNQ3* (Ca). Representative current traces from single oocytes of the same batch injected with the channels mRNA alone or with syntaxin 1A mRNA (0.75 ng/oocyte; +syx). Inset: the voltage protocol used to elicit currents. B, Syntaxin 1A reduces the maximal conductances of KCNQ2 (Ab), and KCNQ2\3 (Bb), but not of KCNQ3* (Cb). Conductance-Voltage (G–V) relationships for the channels in the presence and absence of syntaxin 1A, normalized to the maximal conductance in the absence of syntaxin 1A or each normalized to itself (inset). G values were obtained from peak currents, assuming a reversal potential of −98 mV for K+ ions. Two component Boltzmann equation G/G_max_ = 1/(1+exp(−(V_1/2_−V)/a), ,was fitted to the data. C, Syntaxin 1A slows down only the rate of activation of KCNQ2 (Ac) but not of KCNQ2\3 (Bc) or KCNQ3* (Cc). Inset: overlay of representative traces elicited at +5 mV showing the activation of KCNQ2 currents in the presence and absence of syntaxin 1A. The rising phase of the currents elicited at all denoted potentials was fitted by a bi-exponential function, deriving fast and slow time constants (τ fast and τ slow). Data in B and C were averaged from three oocyte batches with at least 5 oocytes per batch. *p<0.05, **p<0.01.

Next, we assessed the functional consequences of syntaxin 1A binding to the KCNQ2, KCNQ2/3 and KCNQ3 channels by two-electrode voltage clamp analysis of currents evoked by depolarizing potentials in oocytes coexpressing the subunits with syntaxin 1A. However, whereas oocytes expressing KCNQ2 alone or together with KCNQ3 exhibited large outward non-inactivating potassium currents, oocytes expressing KCNQ3 alone did not exhibit any detectable current, in agreement with previous studies [5], [48]–[50]. To investigate the functional impact of syntaxin 1A on KCNQ3 channels, KCNQ3 subunits carrying an A315T mutation in the inner vestibule (KCNQ3*) were expressed instead, evoking measurable currents [51]. The effects of syntaxin 1A on homomeric KCNQ2 channels resulted in both decreased current amplitudes (with no effect on the voltage dependence of activation; Fig. 2) and slower activation kinetics with increased fast (τ_fast_) and slow (τ_slow_) time constants, as derived from fitting with an exponential two-component Boltzmann function (Fig. 2). Whereas the current amplitudes of the KCNQ2 and KCNQ2/3 channels were reduced by syntaxin 1A, those of the KCNQ3* channel were not affected. The effect on activation kinetics was only apparent in KCNQ2 and not in KCNQ2/3 or KCNQ3*. Neither half-activation voltage (V*a*_1/2_) nor the slope factor was affected by syntaxin 1A in all channels. In addition, syntaxin 1A had no effect on deactivation kinetics of the channels (data not shown). It should be noted that both the functional and physical interactions were studied with syntaxin 1A expressed at 0.75 ng/oocyte and lower mRNA concentrations; concentrations that were twice as large had a small impact on KCNQ3 currents (not shown).

Notably, a clear correlation between the physical (Fig. 1) and functional (Fig. 2) interactions of syntaxin 1A emerged from the analysis of the various subunit compositions: KCNQ2 homomers were the most sensitive to syntaxin 1A, both functionally and physically, whereas KCNQ2/3 heteromers were less receptive and KCNQ3 homomers were practically refractory to syntaxin 1A. It should be noted that co-injecting KCNQ2 and KCNQ3 results in the expression of tetrameric channels with various subunit compositions. Therefore, it is possible that syntaxin 1A affected only homomeric KCNQ2 channels within a mixture that consisted of high percentage of KCNQ2/3 channels.

#### Syntaxin 1A does not impair channel synthesis or surface expression

**Figure 3:**
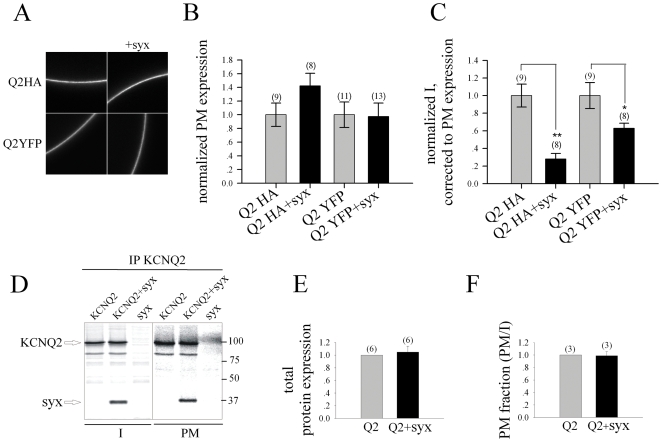
Reduction of currents by syntaxin is not associated with either total channel expression or plasma membrane (PM) content. A, The amount of KCNQ2 (*Q2*) channel in PM is not affected by coexpression of syntaxin (*syx*). Data were obtained by measurements of confocal images in whole oocytes expressing KCNQ2 channel with external HA tag (obtained with an anti-HA antibody) or YFP tag, as indicated, alone or together with syntaxin. B, Summary of KCNQ2 PM expression and comparison of the two imaging methods. Gray bars show PM amount of KCNQ2-HA/YFP channels expressed alone. Black bars show the amount of KCNQ2-HA/YFP coexpressed with syntaxin. In both methods the PM expression level in the presence of syntaxin was normalized to the control group of oocytes expressing the channel alone. Numbers above lanes indicate the numbers of oocytes. C, The effect of coexpression of syntaxin on currents (I), corrected to the corresponding PM expression of KCNQ2-HA or KCNQ2-YFP, was measured from the same oocytes as in A. Currents were evoked by a voltage step from a holding potential of −95 mV to +5 mV and normalized to the control group of oocytes expressing the channel alone. Numbers above lanes indicate the numbers of oocytes. *p<0.05, **p<0.01. D, Syntaxin (*syx*) affects neither total protein expression nor PM content of KCNQ2. Digitized Phosphorimager scan of SDS-PAGE analysis of [^35^S] Met/Cys-labeled KCNQ2 and syntaxin proteins, immunopurified from 110 plasma membranes (right panel; *PM*) or 10 internal fractions (left panel; *I*) of oocytes precipitated by KCNQ2 antibody. KCNQ2 was expressed alone (*KCNQ2*) or together with syntaxin (*+syx*) and protein samples were analyzed on an 8% gel. E, Histogram showing normalized amount of KCNQ2 (*Q2*; quantifies by ImageQuant) expressed with or without syntaxin, precipitated with KCNQ2 antibody from internal fractions (*I*) of oocytes. F, Histogram showing ratios (quantified by ImageQuant) of KCNQ2 amounts in plasma membranes versus internal fractions in oocytes expressing KCNQ2 alone or together with syntaxin, in the same experiments. Numbers above lanes indicate the numbers of experiments.

The reduced amplitudes in oocytes coexpressing syntaxin 1A can arise from effects on either channel surface expression or single-channel conductance and/or open probability. Surface expression is dependent on total channel expression, surface-trafficking efficiencies and/or stability. First, we set out to monitor surface expression of KCNQ2 in oocytes of which current amplitudes were measured beforehand. Plasma membrane (PM) levels were measured by confocal imaging of both KCNQ2-HA channels containing an extracellular HA tag ([52]; using anti-HA antibody) and KCNQ-YFP channels in oocytes expressing the channels with and without syntaxin 1A. Both methods provided very similar assessment of PM expressions, indicating that syntaxin 1A did not impair KCNQ2 PM levels (Fig. 3A,B). Notably, in the same oocytes syntaxin 1A did reduce KCNQ2 current amplitudes (Fig. 3C), suggesting that the reduction of amplitudes is not due to reduced PM levels of KCNQ2. Next, we set out to further asses this notion and to test the effect of syntaxin 1A not only on PM levels but also on total protein expression, surface trafficking efficiencies and stability. To this end we performed an immunoprecipitation analysis of the KCNQ2 content in manually dissected plasma membranes (*PM* fraction) versus that in the cytoplasm+intracellular organelles (*I* fraction) of the same oocytes, in oocytes expressing wild-type KCNQ2 with or without syntaxin 1A (Fig. 3D). In three such experiments, analysis of KCNQ2 expression levels in the *I* fraction of oocytes expressing the channel with or without syntaxin 1A showed similar levels in both groups (Fig. 3D), indicating that total channel expression was not impaired by syntaxin 1A. Furthermore, the PM fraction of KCNQ2, calculated as the intensity ratio of channel content in *PM* versus *I*, was similar in the presence or absence of syntaxin 1A (Fig. 3E). In all, using three different experimental approaches, we determined that neither total channel expression nor cell surface trafficking and stability were impaired by syntaxin 1A and were not the cause of the syntaxin 1A-induced reduction in KCNQ2 macroscopic currents.

#### Mapping the channel domain(s) involved in the syntaxin 1A interaction

Coimmunoprecipitation analysis of syntaxin 1A's association with different chimeric channels, which contained different parts of the KCNQ2 transmembrane segment on the backbone of KCNQ3, showed that the transmembrane segment is not a target for syntaxin 1A-binding and modulation (Figure S1). These findings put forward the intracellular **N- and C- termini of KCNQ2 as potential structural determinants that are critical for syntaxin 1A interaction, both physical and functional**.

**Figure 4:**
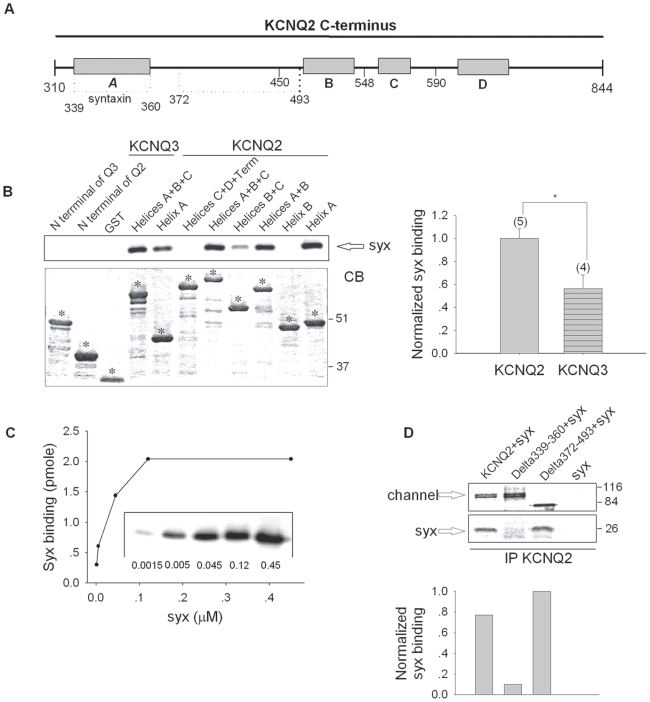
Syntaxin 1A binds preferentially to helix A in the C-terminus and is pivotal for the binding. A, Schematic representation of the C-terminus of KCNQ2, in which the syntaxin 1A (*syx*) binding domain is indicated. B, *Top*: Interaction of syntaxin 1A with GST fusion proteins corresponding to different parts of the C-termini of KCNQ2 and KCNQ3. In vitro synthesized ^35^S labeled syntaxin 1A was reacted with the indicated GST fusion proteins. *Bottom*: Coomassie blue (CB) staining of the protein gel. Numbers denote molecular weight markers. The bar diagram shows the normalized syntaxin 1A binding values. The intensity of the immunoreactive band of syntaxin 1A (*syx*) in different groups was normalized to the corresponding intensity of the coomassie blue (*CB*) staining of the peptide. The data were averaged from several independent experiments and includes the binding of syntaxin 1A to helices A, A+B and A+B+C. C, *Top*: Stochiometry of the binding of syntaxin 1A to helix A of KCNQ2, derived from binding curves that show saturation. Recombinant hexahistidine-tagged (His_6_) cytoplasmic part of syntaxin 1A at the indicated concentrations was bound to immobilized GST-helix A (150 pmol) in a 1 ml reaction volume. Bound syntaxin 1A was determined by SDS-PAGE and immunoblotting with syntaxin 1A antibody (inset). ECL signal intensities were quantified with TINA software and converted to picomoles by the use of standard curves for the corresponding proteins. *Bottom*: Calibration gel which demonstrates the amount of syntaxin 1A coprecipitated in the experiment. Unbound recombinant hexahistidine-tagged (His_6_) cytoplasmic part of syntaxin 1A at the indicated concentrations was loaded on an 8% gel and immunoblotted with syntaxin 1A antibody. D, helix A (aa 339–360) is required for syntaxin 1A's binding to KCNQ2. Oocytes were injected with syntaxin 1A mRNA alone or co-injected with syntaxin 1A and KCNQ2/Δ339–360 deletion mutant/Δ372–493 deletion mutant. The binding assay was performed as described.

To probe for the involvement of N- and C- termini, we started with an *in-vitro* binding assay using immobilized glutathione S-transferase (GST) fusion proteins corresponding to parts of the N and C termini of both KCNQ2 and KCNQ3, and ^35^S-labeled full-length syntaxin 1A, synthesized in reticulocyte lysate. The long (〉500 amino acids) C termini harbor four regions with high probability of forming an α-helical structure (helices A–D) (Fig. 4A). As shown in Figure 4B, syntaxin 1A bound to both channels, but preferentially to channel domains that included helix A (aa 310–450 in KCNQ2 and aa 350–458 in KCNQ3, which share 46% identity); the binding to KCNQ2 was somewhat stronger than to KCNQ3 (Fig. 4B). Interestingly, syntaxin 1A also bound a tandem of helices B+C of KCNQ2 (albeit to a much lesser extent than helix A), although it did not interact with helix B alone. Although one cannot rule out the possibility that other regions of the channel also interact with syntaxin 1A, helix A appears to be sufficient to anchor syntaxin 1A. The binding of syntaxin 1A to helix A of KCNQ2 was further evaluated by using different concentrations of a recombinant hexahistidine (His_6_)-tagged cytoplasmic part of syntaxin 1A. Under our binding conditions, the binding was half-maximal at ∼0.025 µM syntaxin 1A, and at a saturating concentration of syntaxin 1A, the ratio of the binding was 1∶2 (∼2 pmol of syntaxin 1A bound per 4 pmol of helix A) (Fig. 4C). We concluded that the *in-vitro* binding of syntaxin 1A to KCNQ2 is targeted to a defined region, strong, dose-dependent and saturable.

We further aimed to establish the role of helix A in the KCNQ2-syntaxin 1A interaction in oocytes by performing a coimmunoprecipitation analysis with two KCNQ2 deletion mutants, one lacking helix A itself (L339-W360) and the second lacking a dispensable stretch that separates helix A from helix B (L372-W493), which served as a control. Syntaxin 1A bound strongly to KCNQ2 and Δ372–493 deletion mutant, but did not bind Δ339–360 deletion mutant at all and therefore figure 4D clearly shows that helix A is crucial for the binding. Unfortunately, since helix A is also critical for the binding of CaM [20], [21], which regulates surface trafficking of KCNQ2 channels [53], the helix A deletion mutant did not express any current.

#### Colocalization of KCNQ2 and syntaxin 1A is concentrated at synaptic sites in cultured rat hippocampal neurons

**Figure 5:**
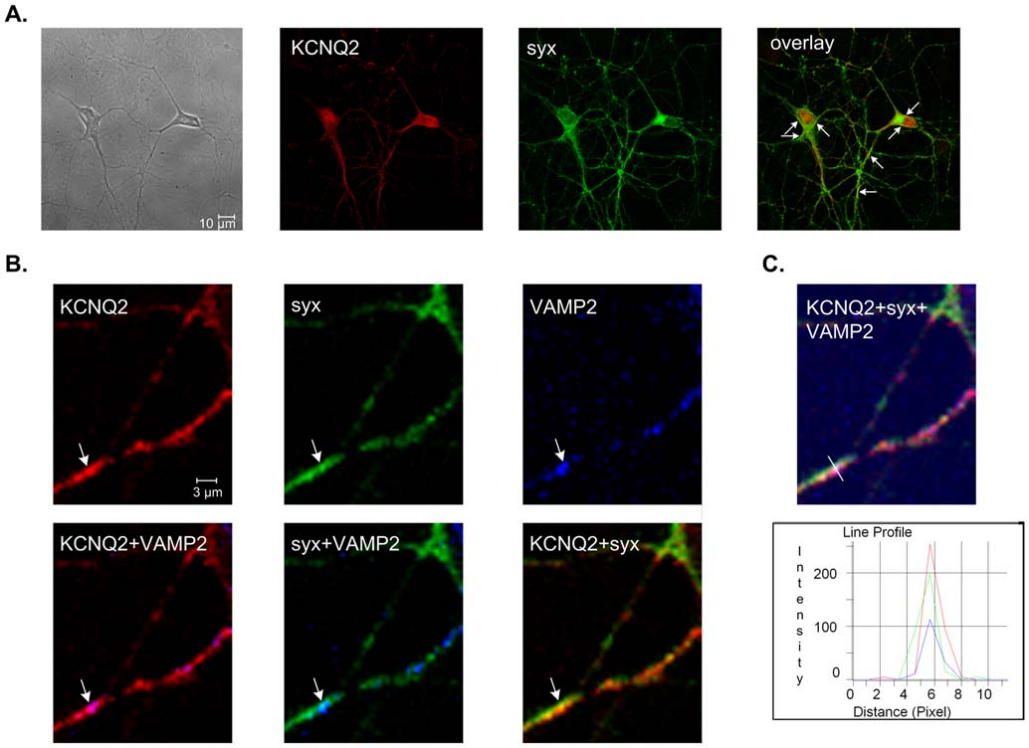
Colocalization of KCNQ2 and syntaxin 1A at synaptic sites marked by VAMP-2 immunoreactivity in hippocampal neurons. A, Immunocytochemistry experiments show colocalization (overlay, yellow) of KCNQ2 (red) and syntaxin 1A (*syx*; green) in rat hippocampal neurons. High colocalization areas of KCNQ2 and syntaxin 1A are indicated by arrows. B, Colocalization of KCNQ2, syntaxin 1A and VAMP-2 in rat hippocampal neurons as detected by triple immunocytochemistry and illustrated by the merge images. KCNQ2 (red), syntaxin 1A (green) and VAMP-2 (blue) are indicated in the top images from left to right. The bottom images from left to right show the colocalization of KCNQ2 and VAMP-2 (merge, pink), syntaxin 1A and VAMP-2 (merge, light blue) and KCNQ2 and syntaxin 1A (merge, yellow). A varicosity colocalized with VAMP-2, syntaxin 1A and KCNQ2 is indicated by arrow. C, The same image as in B showing all three markers; KCNQ2 (red), syntaxin 1A (green) and VAMP-2 (blue). A linescan was placed through the varicosity indicated by arrow in B. The varicosity was shown to colocalize all three signals and thus, is indeed a synaptic one.

Since our results suggested a strong interaction between syntaxin 1A and KCNQ2 subunits, we checked whether the two proteins colocalize in dissociated cultures of hippocampal neurons. Confocal double-staining immunofluorescence microscopy, using antibodies against KCNQ2 and syntaxin 1A, showed staining for KCNQ2 proteins and an apparent high colocalization of syntaxin 1A both in the somata and along the neuronal processes (Fig. 5A). Indeed, quantification of KCNQ2 and syntaxin 1A colocalization yielded a Pearson's correlation coefficient of 0.74±0.01 (13 images were quantified from two different cultures). Next, we determined whether KCNQ2 and syntaxin 1A are colocalized at synaptic sites. We performed a triple-staining immunofluorescence assay, using an additional antibody that recognizes VAMP2 (an integral protein of the vesicular membrane): puncta stained for VAMP2 indicate axonal presynaptic varicosities (Fig. 5B; [54]). We looked for VAMP2-positive puncta colocalizing with puncta positive for both KCNQ2 and syntaxin 1A, and not those colocalizing with the uniformly KCNQ2- or syntaxin 1A-stained somata or processes. Indeed, colocalized KCNQ2+syntaxin 1A appeared to be concentrated at synaptic sites marked by VAMP2 immunoreactivity (Fig. 5C). Quantitative analysis revealed that 53.5±10.8% of VAMP2- and syntaxin 1A-positive varicosities were also positive for KCNQ2 (six images were quantified).

#### KCNQ2 associates with syntaxin 1A in cortical and hippocampal synaptosomes

**Figure 6:**
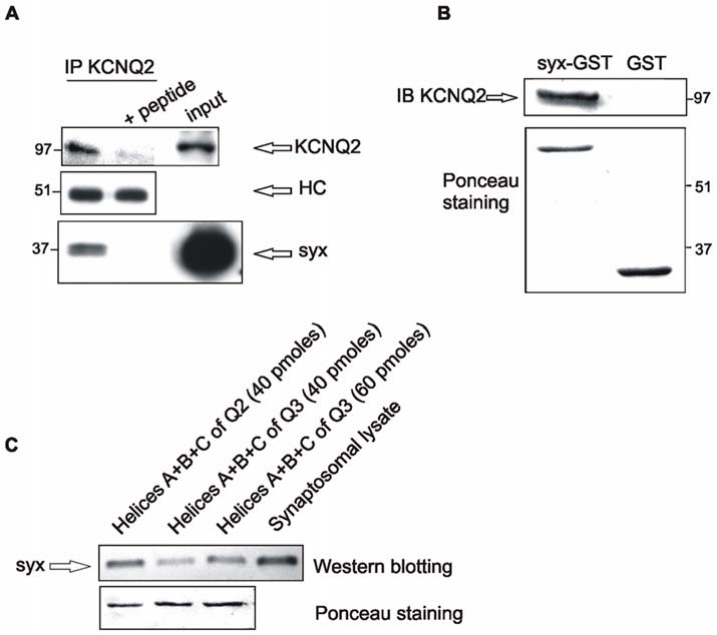
Syntaxin 1A associates with KCNQ2 in cortical and hippocampal synaptosomes. A, Syntaxin 1A (*syx*) coprecipitates with KCNQ2 from 2% Chaps synaptosomal lysate by anti-KCNQ2 antibody. Lysates were incubated with KCNQ2 antibody in the absence or presence (+peptide) of antigen peptide-HC (heavy chain). Numbers indicate molecular weight markers. B, GST-syntaxin 1A fusion protein “pulls down” KCNQ2 from synaptosomal lysates. Syx-GST (corresponding to the cytosolic part of syntaxin 1A) or GST immobilized on GSH-agarose beads (each at 150 pmoles) were incubated with 2% CHAPS lysate (200 µg) for 12 h at 4°C. Precipitated proteins were separated by SDS-gel (8% polyacrylamide) and immunoblotted with anti-KCNQ2 antibody (upper panel). The lower panel shows a Ponceau S staining of the blot, which demonstrates the equal protein loading of syntaxin 1A-GST and GST proteins. C, The binding of syntaxin 1A to helices A+B+C of KCNQ2 is stronger than its binding to the same helices in KCNQ3. Syntaxin 1A coprecipitated with KCNQ2 and KCNQ3 from 2% Chaps synaptosomal lysate by anti-KCNQ2/KCNQ3 antibody. Precipitated proteins were separated by SDS-gel (8% polyacrylamide) and immunoblotted with anti-syntaxin 1A antibody.

Follow-up experiments to further evaluate the interaction between syntaxin 1A and KCNQ2 at presynaptic terminals were carried out using two different assays in rat cortical and hippocampal synaptosomes. First, coimmunoprecipitation analysis using an antibody against KCNQ2, showed that brain syntaxin 1A precipitates with brain KCNQ2. The specificity of this interaction was verified by using KCNQ2 antibody that was preincubated with the peptide against which it was raised: as a consequence, syntaxin 1A precipitation was blocked (Fig. 6A). The second assay was a pull-down assay of synaptosomal KCNQ2 using immobilized GST-syntaxin 1A (corresponding to the cytosolic part of syntaxin 1A). The specificity of this interaction was verified using immobilized GST alone, which did not pull **down any KCNQ2 (** **Fig. 6B** **).**

In addition, we compared the binding of brain syntaxin 1A to KCNQ2 and KCNQ3 in a pull-down assay from synaptosomes (Fig. 6C). Thus, brain syntaxin 1A, similar to *in-vitro*-synthesized syntaxin 1A (Fig. 4B), bound the GST-fused protein corresponding to a tandem of helices A+B+C of KCNQ2 more strongly than that corresponding to KCNQ3.

#### Simulation of syntaxin 1A interaction in a firing neuron

**Figure 7:**
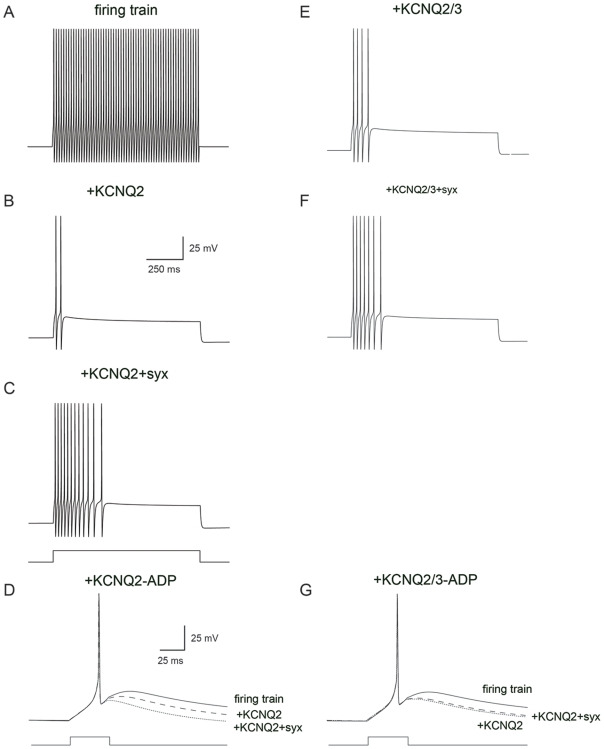
Simulating the effect of the interaction of syntaxin 1A, KCNQ2 and KCNQ2/3 on neuronal physiology. A, A regular firing train of action potentials generated by a 2 nA current injection into a spherical neuron containing the Hodgkin-Huxley model (current step is shown below panel c and is similar for A, B, and C). B, Similar simulation to a containing, in addition to the Hodgkin-Huxley model, also a model of the KCNQ2 as described in the *Materials and Methods* and Table 1 at a density of 5 pS/µm^2^. The scale bar in B applies also to A, C, E, F, and G. C, Similar simulation to a containing, in addition to the Hodgkin-Huxley model, also a model of the KCNQ2+syntaxin 1A (*syx*) as described in the *Materials and Methods* and Table 1 at a density of 2.5 pS/µm^2^. D, Simulation of the effect of KCNQ2 on the action potential ADP in a single compartment model containing the Hodgkin-Huxley model and a T-type voltage-gated Ca^2+^ conductance (smooth line). Adding 5 pS/µm^2^ KCNQ2 to the ADP model reduced the amplitude of the ADP (dotted line). Simulating the effect of the interaction of syntaxin 1A with KCNQ2 by using the relevant time constants from Table 1 and halving the maximal conductance generated a larger ADP (dashed lines). The scale bar in D applies also to H. E, Same as in B only with KCNQ2/3. F, Same as is c only with KCNQ2/3.

To investigate the possible physiological impact of the interaction of syntaxin with KCNQ2 and KCNQ3 we performed numerical simulations. A kinetic model of KCNQ2 conductance added to the regular firing Hodgkin-Huxley model (Fig. 7A) generated, as predicted for the M-channel conductance, substantial spike frequency adaptation (Fig. 7B). It is important to note that our modeling was designed to qualitatively investigate the physiological impact of the interaction of syntaxin with KCNQ2 and KCNQ3. Thus, while reproducing the general effect it cannot be used for quantitative analysis. The interaction of syntaxin 1A with KCNQ2 prolonged the activation time constants of this conductance and reduced the conductance of the current roughly twofold (Fig. 2). Therefore, this slowed activation could be predicted to reduce spike frequency adaptation relative to the KCNQ2 conductance in the absence of syntaxin 1A interaction. Indeed, when we replaced the kinetics of KCNQ2 with the kinetics of KCNQ2+syntaxin 1A in the model and halved the conductance density, the simulated neuron generated more action potentials for the same current injection (Fig. 7C).

Recently the M-channel has been implicated in control of the action potential after depolarization (ADP) [55], [56]. To investigate the effect of KCNQ2 on the ADP, we added T-type voltage-gated Ca^2+^ conductance—to simulate ADP in the model—to the regular firing Hodgkin-Huxley model (Fig. 7D). Increasing the conductance of the KCNQ2 model reduced the ADP considerably (Fig. 7D). Replacing the KCNQ2 model with that of KCNQ2+syntaxin 1A and halving the conductance density produced a larger ADP than that simulated in the presence of KCNQ2 alone (Fig. 7D).

Next, we turned to simulating the effect of KCNQ2/3 on spike frequency adaptation and on ADP. The activation time constant for KCNQ2/3 was slower than that for KCNQ2 (Table 1), and thus, more action potentials were generated in the presence of a similar conductance density (Fig. 7E) than with KCNQ2 (Fig. 7B). The activation time constants of KCNQ2/3 were not affected by the interaction with syntaxin 1A and the total conductance was reduced by only 25% (Fig. 2). When the conductance density of the KCNQ2/3 model was reduced by 25%, more action potentials were generated for the same current injection (Fig. 7F) but the decrease in spike frequency adaptation was not as substantial as in the model of KCNQ2+syntaxin 1A (Fig. 7C). Finally, we investigated the possible effect of the interaction of KCNQ2/3 with syntaxin 1A on the ADP. Similar to KCNQ2, KCNQ2/3 reduced its amplitude. When the maximal conductance of KCNQ2/3 was reduced by 25%, to simulate the association with syntaxin 1A, there was only a small effect on the amplitude of the ADP and not as substantial as in the model of KCNQ2+syntaxin 1A (Fig. 7D).

## Discussion

Along with their involvement in the fusion process, the SNARE proteins have been shown to interact directly with ion channels. To date, a number of potassium channels belonging to the Kv1, Kv2 and Kv4 families of the Kv superfamily have been recognized to be regulated by SNARE proteins [27]–[31], [46], [57]–[60]. Specifically, syntaxin 1A interacts with distinct domains, on either the N or C cytosolic termini, within the different Kv members and induces distinct functional modulations that include diverse effects on activation and inactivation gating and trafficking of the channels [61].

The present study establishes a novel interaction between syntaxin 1A and a specific member of the Kv7 family, KCNQ2, but not KCNQ3 or KCNQ1, which bind syntaxin 1A more weakly (Fig. 1B,C). Here, we demonstrated that syntaxin 1A colocalizes with KCNQ2 subunits at hippocampal presynaptic boutons (Fig. 5A), binds to KCNQ2 in both synaptosomal membranes (Fig. 6C) and oocytes (Fig. 1A,B), and modulates the function of the homomeric KCNQ2 channels expressed in oocytes (Fig. 2) by slowing the activation rate ∼2 fold and decreasing the current amplitudes by about 50%; the latter is not due to reduced total expression or cell surface trafficking and stability of the channels (Fig. 3). Notably, a clear correlation between the physical (Fig. 1) and functional (Fig. 2) interactions of syntaxin 1A emerged from the analysis of channels with various subunit compositions. Heteromeric KCNQ2/3 channels, which bind syntaxin 1A to a smaller extent, were less sensitive to syntaxin 1A's action: their activation kinetics were not affected at all and their amplitudes were decreased by only 20% (Fig. 2). KCNQ3 homomeric channels, which bound syntaxin 1A weakly as compared to that of homomeric KCNQ2 channels, were practically resistant to syntaxin 1A. Consequently, homomeric KCNQ2 channels are the primary target of syntaxin 1A modulation.

Importantly, the selective and robust syntaxin 1A-induced impact on the activation gating of KCNQ2 compared to KCNQ2/3 may be of physiological significance. KCNQ2 and KCNQ3 channels are expressed at different subcellular locations, including somatodendritic, axonal and terminal sites. This multifaceted subcellular distribution of KCNQ2/3 channels enables them to be involved in both pre- and postsynaptic modulation of neurotransmission. KCNQ2 and KCNQ3 subunits are found to be coexpressed at the nodes of Ranvier and at the axon initial segments of several central and peripheral neurons [8], [9], [62]. Many axon initial segments of pyramidal neurons in hippocampal CA1 and CA3 layers and of temporal neocortex express both KCNQ2 and KCNQ3 subunits. The axon initial segment is a strategic site for M-channels to shape the spike ADP waveform and modulate spike frequency adaptation [55], [56], [63]. Thus, KCNQ2/3 channel activity may influence intrinsic excitability at the initial segment, where fast spikes as well as spike ADPs are initiated [64], [65]. ADP depends on the interplay between persistent sodium currents (I_NaP_), which tend to increase the ADP to the point of bursting, and M-currents, which restrain the ADP and prevent repetitive discharge [55], [56]. Thus, a very slow M-current (KCNQ2/3) activation would not be able to prevent the escalation of the ADP into a spike burst (demonstrated in Fig. 7).

In contrast, a selective robust slowing and reduction of KCNQ2 activation at presynaptic terminals would be nicely shaped to modulate the release of neurotransmitters. Several recent studies have indicated that activation of M-channel with openers inhibits the release of dopamine *in vitro* and *in vivo* [66], [67]. M-channels have been shown to play an important role in the presynaptic control of dopamine (DA) release from striatal nerve endings induced by direct membrane depolarization. Blocking of M-channels has been found to enhance striatal release of catecholamines and to reinforce the depolarization-induced DA release evoked by presynaptic muscarinic receptor activation [67]. Presynaptic M-channels have been suggested to regulate neurotransmitter release in hippocampal synaptosomes preloaded with [^3^H]noradrenaline, [^3^H]GABA and D-[^3^H]aspartate [68]. More recently, it was found that activation of M-channels by a channel opener decreases the frequency of miniature excitatory and inhibitory postsynaptic currents (mEPSC and mIPSC, respectively) without affecting their amplitude or waveform, thus suggesting that M-channels presynaptically inhibit glutamate and GABA release [69]. Also, it was shown that M-channels modulate the release of noradrenaline in superior cervical ganglion neurons [70]. Notably, KCNQ2 but not KCNQ3 subunits have been suggested to play a major role in the regulation of neurotransmitter release [68].

A C-terminal syntaxin 1A-association domain in KCNQ2, helix A, was pinpointed by analysis of different KCNQ2 and KCNQ3 chimeras and deletion mutants, combined with *in-vitro* binding analysis. The strong *in-vitro* binding of syntaxin 1A (Fig. 4B) and the inability of syntaxin 1A to associate with KCNQ2 lacking helix A in oocytes (Fig. 4D), gave support to the notion that helix A is a crucial region for KCNQ2-syntaxin 1A binding. However, the *in-vitro* binding potencies of helix A in KCNQ2 and KCNQ3 were not that different (Fig. 4B), and probably do not account for the different syntaxin 1A-binding potencies of the respective channels in oocytes (Fig. 1A,C). Therefore, we can only hypothesize that the target site in KCNQ2 for syntaxin 1A may be comprised of parts additional to helix A, such as the tandem of helices B+C that could bind syntaxin 1A *in vitro* to some extent (Fig. 4B); together with helix A, these might create an optimal binding pocket for syntaxin 1A, which may not exist in KCNQ3. Alternatively, the syntaxin 1A binding pockets may be similar in the KCNQ2 and KCNQ3 channels but differ in their interaction with other parts of the channels, which may interfere with the interaction of syntaxin 1A with helix A by preventing appropriate access to this site.

The binding of syntaxin 1A to KCNQ2 affected channel function without altering plasma membrane channel density (*N*; Fig. 3), suggesting that single channel open probability (*Po*) and/or conductance (*γ*) were affected. Indeed, we have previously demonstrated that the binding of syntaxin 1A to the cytoplasmic N tail of another Kv channel, Kv1.1 affects both *Po* and *γ* (Michaelevski 2007). The question arising is the molecular mechanism by which syntaxin 1A binding to Helix A at the cytoplasmic C tail causes allosteric conformational changes in the gating and/or pore machineries. In this regard, it has been shown that PIP_2_ binding to the C terminus at a region following Helix A affects *Po* [71]. It was suggested that this was achieved through exertion of force that pulls the sixth transmembrane segment (S6), which contains the main gate of Kv channels [72], relatively to the pore [73]. Similar mechanism could apply also for syntaxin 1A effect on KCNQ2.

However, because the *in vitro* binding of KCNQ3 were not significantly different from those of KCNQ2, and KCNQ3 still bound syntaxin 1A *in vivo* (albeit weakly as compared to KCNQ2), it may well be that the differences in syntaxin 1A functional potencies between KCNQ2 and KCNQ3 may not be due to the differential binding affinities, but rather to differential coupling efficiencies between syntaxin 1A binding domain and the gating and/or conductance machinery. This suggests involvement of different intramolecular interactions in the function of syntaxin 1A on the channels. Similar considerations were suggested to account for the differences in PIP2 efficacies between KCNQ2 and KCNQ3 [73]. We favor the idea of involvement of interdomain interactions between the N and C tails for the following two reasons: i) there is a body of evidence for N/C interactions as a mechanism for the regulation of Kv channel gating **(**e.g., [74]–[79]; ii) we have previously suggested that the differences between the N termini of KCNQ2 and KCNQ3 account for their different *Po* values [51], [80].

Remarkably, Helix A forms a critical part of the KCNQ2 Ca^2+^-CaM interaction site [17], [20], [21], [81]–[83], which is critical not only for channel surface expression [53], [84], but also for a voltage-independent regulation of channel *Po* [80]. This suggests a possible interplay between the modulations by syntaxin 1A and Ca^2+^-CaM, resulting from either mutually exclusive or, vice-versa, synergistic bindings of the two proteins. Mutually exclusive binding of Ca^2+^-CaM and other proteins to a common binding domain have been shown to play a role in the regulation of NMDA (N-methyl-D-aspartic acid) receptors, TRP (Transient Receptor Potential) channels and metabotropic glutamate receptors. Thus, Ca^2+^-CaM and Gβγ bind to partially overlapping domains located in the N-terminal part of the mGluR 7 C-tail, and mutations that prevent Ca^2+^-CaM binding selectively inhibit mGluR 7 signaling through Gβγ subunits but do not affect trimeric G-protein recruitment to the receptor [85]. Common binding sites for Ca^2+^-CaM and inositol 1,4,5-trisphosphate receptors [IP(3)Rs] have been identified on the C termini of TRP channels [86]. Ca^2+^-CaM can also compete for a common binding site on NR1 (NMDA receptor 1) with myosin RLC (regulatory light chain) [87]. In addition, our lab reported the binding of syntaxin 1A and Gβγ to partially overlapping domains at the N terminus of the voltage-gated potassium channel Kv1.1, forming a complex which plays a role in the modulation of Kv1.1 inactivation [47]. Thus, it remains possible that Ca^2+^-CaM and syntaxin 1A, and possibly other signaling molecules, interact with KCNQ2 subunits at sequences overlapping or adjacent to helix A, mutually affecting their binding and hence channel function.

Taken together, this study suggests that the interaction of KCNQ2 homomeric channels with syntaxin 1A may play a role in the regulation of presynaptic vesicle release, similar to that of the Kv2.1-syntaxin 1A interaction in neuroendocrine dense-core vesicle release [32], [33].

## Supporting Information

Figure S1 The transmembrane segments do not confer syntaxin the ability to bind the channels. a, Schematic representation of the chimeras. The boxes indicate transmembrane segments and the loop represents the pore between S5 and S6. Segments from KCNQ2 are shaded in black and those from KCNQ3 in white. b, Digitized Phosphorimager scan of SDS-PAGE analysis of [35S] Met/Cys-labeled channels, chimeras and syntaxin (syx) proteins coprecipitated by the corresponding antibodies from 1% Triton X-100 homogenates of whole oocytes, that were injected with the channels/chimeras mRNA alone or coinjected with syntaxin mRNAs (as indicated above the lanes). c, syntaxin affected neither the current amplitudes (upper panel) nor the time constants of activation (lower panels) of the chimera Q3/Q290-310.
(0.19 MB TIF)
