# Supplementary figures and images for: Selective Interaction of Syntaxin 1A with KCNQ2: Possible Implications for Specific Modulation of Presynaptic Activity
Source: PLoS One. 2009 Aug 13;4(8):e6586. doi: 10.1371/journal.pone.0006586 (PMC2721677; doi:10.1371/journal.pone.0006586)

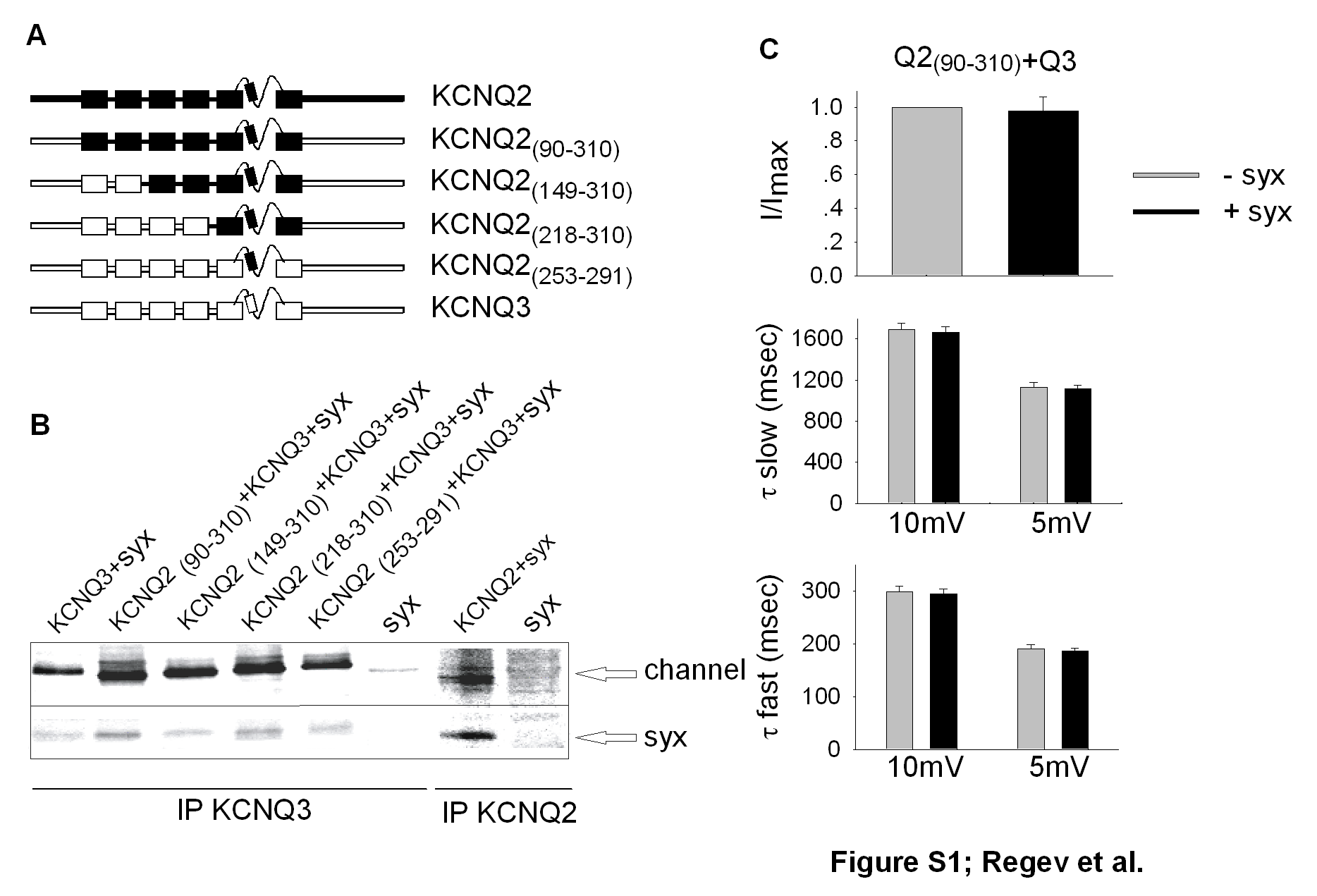

Supplement: Figure S1 — The transmembrane segments do not confer syntaxin the ability to bind the channels. a, Schematic representation of the chimeras. The boxes indicate transmembrane segments and the loop represents the pore between S5 and S6. Segments from KCNQ2 are shaded in black and those from KCNQ3 in white. b, Digitized Phosphorimager scan of SDS-PAGE analysis of [35S] Met/Cys-labeled channels, chimeras and syntaxin (syx) proteins coprecipitated by the corresponding antibodies from 1% Triton X-100 homogenates of whole oocytes, that were injected with the channels/chimeras mRNA alone or coinjected with syntaxin mRNAs (as indicated above the lanes). c, syntaxin affected neither the current amplitudes (upper panel) nor the time constants of activation (lower panels) of the chimera Q3/Q290-310. (0.19 MB TIF) [file pone.0006586.s001.tif]
